# Transcriptional regulation of mixed lineage kinase 3 by estrogen and its implication in ER-positive breast cancer pathogenesis

**DOI:** 10.18632/oncotarget.16566

**Published:** 2017-03-25

**Authors:** Navin Viswakarma, Rakesh Sathish Nair, Gautam Sondarva, Subhasis Das, Lucas Ibrahimi, Zhiyong Chen, Subhash Sinha, Basabi Rana, Ajay Rana

**Affiliations:** ^1^ Department of Surgery, Division of Surgical Oncology, University of Illinois at Chicago, Chicago, IL 60612, USA; ^2^ University of Illinois Hospital and Health Sciences System Cancer Center, University of Illinois at Chicago, Chicago, IL 60612, USA; ^3^ Jesse Brown VA Medical Center, Chicago, IL 60612, USA; ^4^ Department of Cell and Molecular Biology, The Scripps Research Institute, La Jolla, CA 92037, USA

**Keywords:** estrogen receptor, MLK3, transcriptional regulation, breast cancer, estrogen response element

## Abstract

Mixed Lineage Kinase 3 (MLK3), also called as MAP3K11 is a tightly regulated MAP3K member but its cellular function is still not fully understood. Earlier we reported post-translational regulation of MLK3 by estrogen (E2) that inhibited the kinase activity and favored survival of ER^+^ breast cancer cells. Here we report that *MLK3* is also transcriptionally downregulated by E2 in ER^+^ breast cancer cells. Publicly available data and *in situ* hybridization of human breast tumors showed significant down regulation of *MLK3* transcripts in ER^+^ tumors. The basal level of *MLK3* transcripts and protein in ER^+^ breast cancer cell lines were significantly lower, and the protein expression was further down regulated by E2 in a time-dependent manner. Analysis of the promoter of *MLK3* revealed two ERE sites which were regulated by E2 in ER^+^ but not in ER^−^ breast cancer cell lines. Both ERα and ERβ were able to bind to *MLK3* promoter and recruit nuclear receptor co-repressors (NCoR, SMRT and LCoR), leading to down-regulation of *MLK3* transcripts. Collectively these results suggest that recruitment of nuclear receptor co-repressor is a key feature of ligand-dependent transcriptional repression of *MLK3* by ERs. Therefore coordinated transcriptional and post-translational repression of pro-apoptotic MLK3 probably is one of the mechanisms by which ER^+^ breast cancer cells proliferate and survive.

## INTRODUCTION

Mixed Lineage Kinase 3 (MLK3) is a member of a larger family of mitogen-activated protein kinase kinase kinase (MAP3K) [1]. MLK3 derives its unique name due to its catalytic domain that contains signature sequences of Ser/Thr as well as Tyr kinases, signifying that the family members of MLK contain mixed characters of both subtypes of kinases [1, 2]. Our group and others have reported that MLK3 indeed is a functional Ser/Thr kinase and directly phosphorylates MKK4 and MKK7 on Ser/Thr to activate downstream JNK [3–5]. However, tyrosine kinase activity of MLK3 is not yet known. The cellular function of MLK3 or any other family member is not fully understood. In dopaminergic neurons, it is reported that activation of MLK3 promotes neuronal loss and leads to neurodegenerative disorder, like Parkinson's Disease [6]. Since cancer is a proliferative disease, in contrast to neurodegenerative disease, our group sought to explore its regulation in cancer, using breast cancer as a model [7].

The hormone receptors, estrogen receptor (ER) and progesterone receptor (PR), and human epidermal receptor 2 (HER2) serve as prognostic and diagnostic markers in breast cancer [8, 9]. These receptors are reported to activate the downstream signaling pathway to promote breast cancer cell survival and growth [9]. Therefore, based on the overexpression of these receptors, breast cancers are classified as ER-positive, where ER expression is several fold above its basal expression level and HER2 positive, where HER2 expression is million fold above the normal. Accordingly, these receptors serve as drug targets and patients are treated with their antagonists [9–11]. These amplified receptors exert their effects by upregulating the expression of genes encoding for proliferation and survival of breast cancer and at the same time, suppressing the genes that promote cell death.

Earlier, we reported that the kinase activity of proapoptotic MLK3 was downregulated by estrogen (E2) in ER+ breast tumors and cell lines [7]. The inhibition of MLK3 kinase activity by E2 was through direct phosphorylation of MLK3 by a pro-survival kinase, AKT; where Ser674 site on MLK3 was phosphorylated by E2-activated AKT, leading to ER^+^ breast cancer cell survival [7]. While performing these experiments, we also observed that MLK3 expression was noticeably lower in ER^+^ compared to ER^−^ breast cancer cell lines. These indicated the possibility that MLK3 expression might be additionally regulated at a transcriptional level by the genomic action of E2-ERs axis.

Here we report that *MLK3* is indeed transcriptionally repressed by E2-ERs axis. We cloned *MLK3* promoter and identified two classical estrogen response elements (EREs) on *MLK3* promoter. The *MLK3* promoter reporter activity was repressed upon E2 treatment in ER^+^ but not in ER^−^ breast cancer cell lines. Additionally, *in-situ* hybridization analysis showed significant upregulation of *MLK3* transcripts in ER^−^ compared to ER^+^ human breast tumors. Concurrently, E2 was able to suppress MLK3 protein expression after 72–120 hours of treatment. All of these collectively indicated a genomic antagonistic action of E2 on *MLK3* promoter. We also identified three corepressors: NCoR, SMRT and LCoR that played a direct role in suppressing *MLK3* transcription through ligand bound ER.

Our study thus provides evidence that ligand bound ER recruits corepressors to inhibit *MLK3* transcription. Since MLK3 is a pro-apoptotic kinase, and we reported that its activity is required for ER^+^ cancer cell death, it seems likely that suppression of *MLK3* transcription could be an additional mechanism by which ER^+^ breast cancer cells evade death for their uncontrolled proliferation and survival.

## RESULTS

### *MLK3* transcripts are downregulated in ER^+^ breast tumors

**Figure 1:**
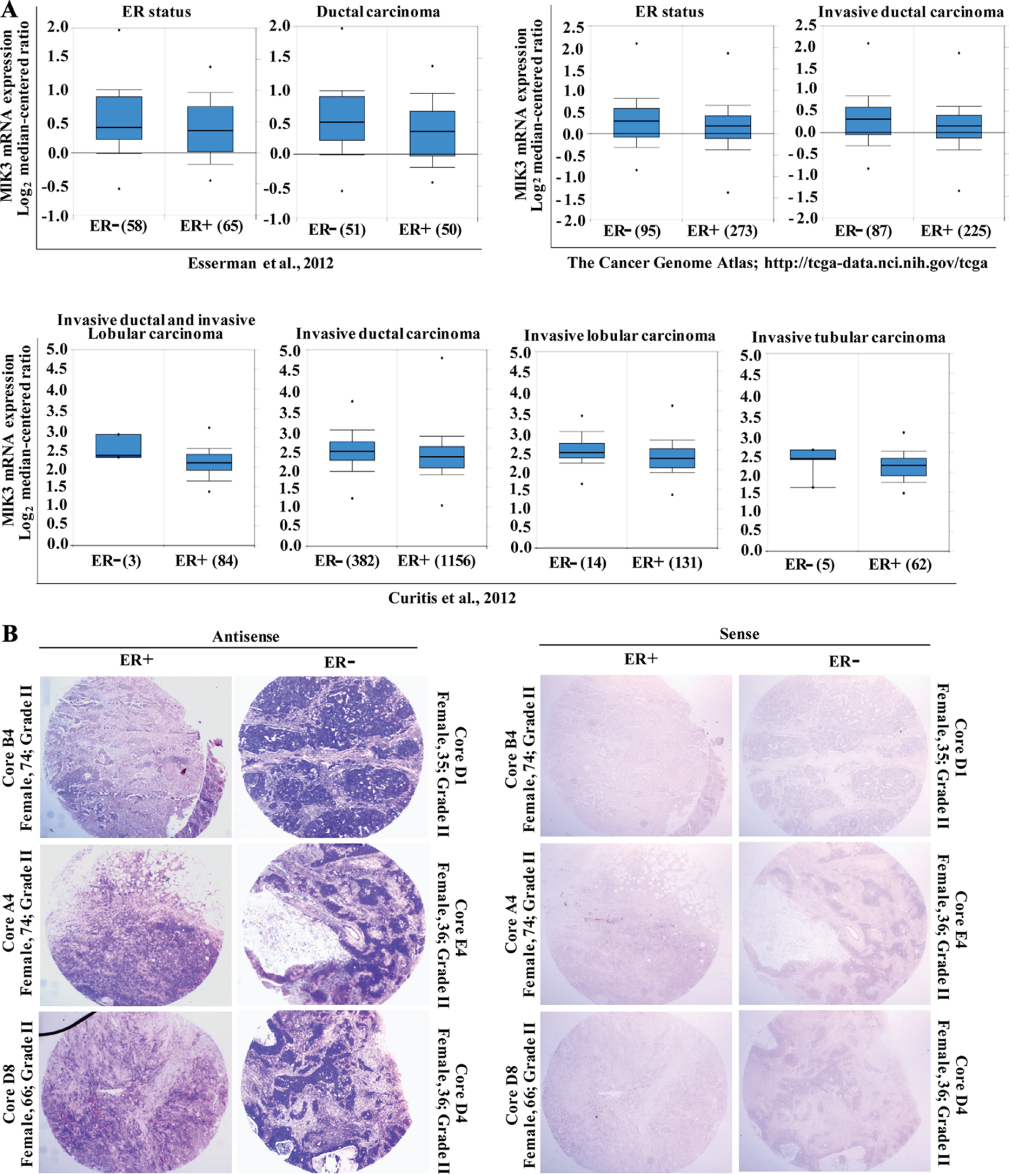
*MLK3* expression is lower in estrogen receptor positive breast cancer tumors (**A**) Three independent data sets were obtained from ONCOMINE (www.oncomine.org). All data are log transformed and median centered (Y-axis). The 25th–75th percentiles are indicated within the closed blue box; the median is indicated by the solid line; the 10th and 90th percentiles are indicated by the bars. Closed circles above and below the plots show sample maximum and minimum values. The number of breast samples present in each group is shown within parentheses. (**B**) *In-situ* hybridization of breast tumor tissues array with *MLK3* antisense and sense riboprobes were performed.

Previously we reported that the kinase activity of MLK3 was significantly lower in ER^+^ as compared to ER^−^ breast tumors [7]. Furthermore, we also reported that MLK3 kinase activity was inhibited by E2 via activation of PI3K-AKT pathway in ER^+^ breast cancer cell lines. Importantly, for these studies, the kinase assays were done taking normalized expression of MLK3 due to differential expression of endogenous MLK3 in different breast cancer cell lines and primary tumors. Interestingly, MLK3 protein expression was lower in ER^+^ breast cancer cell lines, suggesting the possibility that there are additional regulations of *MLK3* (at the level of expression) by E2-ER axis. Given ERs role in the transcriptional regulation of various genes, we first decided to examine the status of *MLK3* mRNA expression in publically available database in ER^+^ and compared with ER^−^ breast tumors. Cancer microarray-based database, ONCOMINE (www.oncomine.org), showed indeed *MLK3* mRNAs were statistically lower in ER^+^ compared to ER^−^ ductal carcinoma (Figure 1A). Three independent data sets: I-SPY 1 population [12], The Cancer Genome Atlas (TCGA) and a high-risk, ER^+^ 11q13/14 cis-acting novel subgroup [13] were further analyzed and all the data sets showed consistent lower *MLK3* transcripts in ER^+^ compared to ER^−^ (Figure 1A). These data suggested that perhaps E2-ER axis downregulates the transcription of *MLK3* gene in breast cancer.

To confirm that *MLK3* transcripts are significantly lower in ER^+^ as compared to ER^−^ breast tumors, we performed *in situ* hybridization on breast tissue microarray, containing ER^+^ and ER^−^ ductal breast carcinomas. The tissue microarray used consisted of total 16 cases of breast cancer, each in duplicates with known receptor status, diagnosis and age of the patients. Hybridization with antisense probe showed intense staining of *MLK3* transcripts in ER^−^ invasive ductal (IDC) and infiltrating tubular carcinomas which was significantly downregulated in ER^+^ tumor cores (Figure 1B). The sense *MLK3* probe did not show any non-specific staining (Figure 1B). The pathology of tumors from ER^+^ and ER^−^ breast cancer patients were confirmed by H&E staining (Supplementary Figure 1A). Interestingly, some of the ER^−^ cores, especially from younger patients with Comedo carcinoma like pathology had significantly higher *MLK3* transcripts (Supplementary Figure 1D). Since Comedo carcinoma is reported to be an early breast cancer lesion [14], our results suggest that induction of *MLK3* expression might represent the initiation of breast cancer. Taken together these results indicated that possibly *MLK3* is transcriptionally downregulated via E2-ER axis.

### *MLK3* transcript and protein are downregulated in ER^+^ breast cancer cell lines

**Figure 2:**
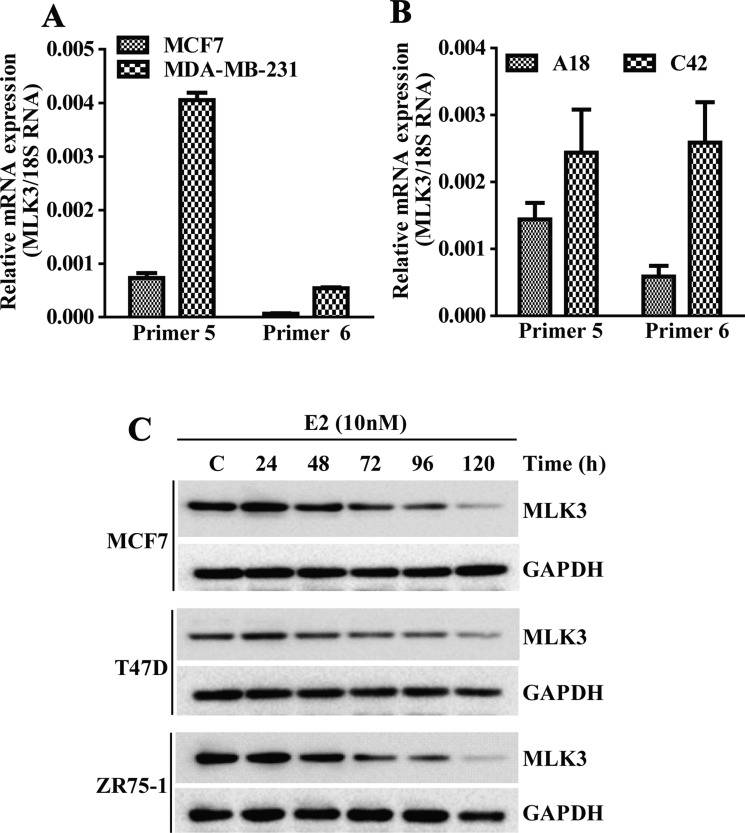
*MLK3* expression is regulated by E2-ER axis: the *MLK3* mRNA expression levels were quantified by Real Time PCR (**A**) *MLK3* mRNA levels in MCF7 (ER+) were compared to MDA-MB-231(ER^−^) cells, using two pairs of specific primer sets. (**B**) *MLK3* mRNA levels in isogenic T47D cell lines: A18; (ER^+^) and C42; (ER^−^) were determined using same set of primers, like in A. (**C**) The ER+ MCF7, T47D and ZR75-1 breast cancer cell lines were treated with E2 (10 nM) in phenol red free medium containing 0.2% charcoal stripped serum for different periods of time as indicated. Lysate were prepared in RIPA buffer and immunoblotted with anti-MLK3 antibody. GAPDH was used as a loading control.

To determine whether *MLK3* is also differentially expressed in established breast cancer cell lines, based on their ER status, we examined basal mRNA level by Real Time PCR in MCF7 (ER^+^) and compared it with MDA-MB-231 (ER^−^) cell lines. Cells were grown in phenol red free, charcoal stripped medium and RNA was converted to cDNA and real time PCR was performed with 2 sets of primers, taking 18S rRNA as a housekeeping control. The *MLK3* transcripts were significantly lower in ER^+^ MCF7 compared to ER^−^ MDA-MB-231 cells lines (Figure 2A). To determine conclusively that E2-ER axis does lead to a downregulation of *MLK3* transcript, we also used a syngeneic breast cancer cell model, where ER was progressively lost in an ER^+^ parental line. T47D:A18 is a ER^+^ breast cancer cell line and its syngeneic clone, T47D:C42 was established as an ER^−^ breast cancer cell line [15]. Our result showed that *MLK3* transcript was significantly lower in parental ER^+^ T47D:A18 cell line, as compared to its syngeneic ER^−^, T47D:C42 clone (Figure 2B). To determine that decreased *MLK3* transcription in ER^+^ breast cancer cell is mediated via E2-ER axis, and diminished mRNA expression ultimately leads to decreased MLK3 protein expression, we treated three ER^+^ breast cancer cell lines (MCF7, T47D and ZR75.1) with physiological dose of E2 (10 nM) for different periods of time (1–5 days). As shown in Figure 2C, MLK3 protein expression was similarly down-regulated in all three ER^+^ breast cancer cell lines upon E2 treatment in a time-dependent manner. The significant decrease in MLK3 protein expression was apparent at 72 hrs. and was maximal at 120 hrs. in all three cell lines (Figure 2C). Since it took at least 72 hrs. to observe any appreciable decrease in MLK3 protein expression, these data suggest that E2-ER axis downregulate MLK3 protein expression via genomic action of ER.

### Identification and cloning of human *MLK3* promoter

**Figure 3:**
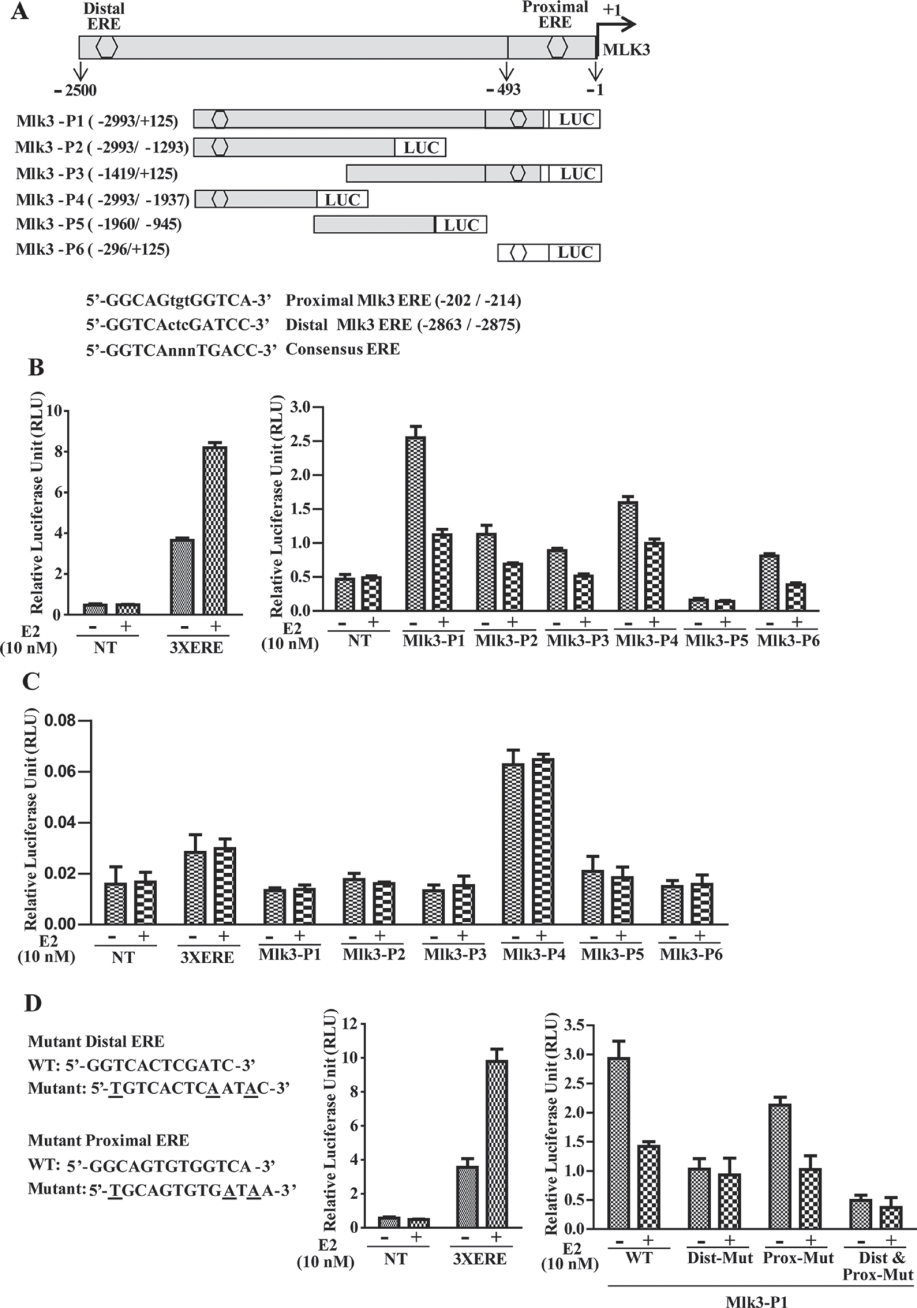
Estrogen Receptor regulates *MLK3* promoter upon E2 treatment (**A**) schematic representation of *MLK3* promoter cloned into pGL4.16 [luc2CP/Hygro] vector. Two putative EREs (hexagon) designated as proximal and distal are shown. Sequence of ERE binding sites with respective consensus sequences are shown below. A series of 5′ and 3′ progressive deletion mutants were generated by nested PCR using MLK3-P1 (−2993/+125) as a template. Five deletions mutants were designated: MLK3-P2 (−2993/−1293), MLK3-P3 (−1419/+125), MLK3-P4 (−2993/−1937), MLK3-P5 (−1960/−945) and MLK3-P6 (−296/+125). Position of EREs is indicated with reference to translation start codon ATG. Promoter activity was assayed in (**B**) ER^+^, MCF7 and (**C**) ER^−^, MDA-MB-231 cell lines as described in Materials and Methods. E2 (10 nM) or ethanol (vehicle) were added to the medium after 14 h of transfection. Cells were harvested 36 h after transfection. Luciferase activities were measured using the Dual Luciferase Assay Kit (Promega). Values are the mean ± S.D. for at least three independent experiments. *p < 0.05; ***p < 0.001 versus ethanol control. (**D**) Mutation in ERE attenuate MLK3 promoter activity. The distal and proximal EREs were mutated either alone, or together (Dist+Prox-Mut) in Mlk3-P1 and luciferase activities were measured as in B and C.

To validate a potential transcriptional regulation of *MLK3* by E2-ER axis, next we asked whether *MLK3* promoter was regulated by ER via direct binding to ERE (Estrogen Receptor Element). We analyzed approximately 3 kb upstream sequence between first ATG (where A is designated as +1) and adjacent gene PCNXL3 located on the chromosome 11 (q13.1-q13.3), using MatInspector software from the Genomatix (version 3.2) [16]. The promoter sequence was submitted to the GenBank (accession no. KP144995). Two putative EREs were identified with significantly high matrix score located at −2863/−2875 bp and −202/−214 bp. These EREs were designated as distal- (−2863/−2875) and proximal- (−202/−214) EREs respectively. A core promoter of 60 bp was predicted using Eukaryotic Promoter Database (http://epd.vital-it.ch/) from the Swiss Institute of Bioinformatics, located −483 to −542 bp that also contains putative RNA pol II binding motif (−499/−505 bp) upstream to the transcription start site. Among all predicted transcription factor binding motifs in the *MLK3* promoter, we focused our efforts on the characterization of the two putative EREs that contain 13 bp imperfect palindrome separated by 3 bp (DR3-like). The nucleotide sequences and positions of these putative EREs are as follows: proximal-ERE: 5′-GGTCActcGATCC-3′ (−202/−214) and distal-ERE: 5′-GGCAGtgtGGTCA-3′ (−2863/−2875) (Figure 3A). The sequence of distal-ERE resembles more closely to the consensus ERE that is present in most of the ER^−^responsive genes (Figure 3A) [17].

### Estrogen treatment decreases *MLK3* promoter activity

To determine the functionality of the putative EREs in the *MLK3* promoter, 3.1 kb genomic fragment was amplified from the HEK293 cells and cloned into the pGL4.16, and designated as MLK3-P1 (−2993/+125; Figure 3A). This amplified region contains 2.5 kb sequence upstream of the transcription start site, 493 bp 5′ UTR and 125 bp from the MLK3 coding sequence. As shown in Figure 3A, a series of 5′ and 3′ progressive deletions were generated by nested PCR using MLK3-P1 as a template, and their activities were measured by their ability to drive luciferase expression in MCF7 (ER^+^) and MDA-MB-231 (ER*^−^*) breast cancer cell lines (Figure 3B and 3C). Nested deletions were created based on positions of ERE and constructs containing either of the EREs, or without EREs were generated (Figure 3A). The 5′ deletions were designated as MLK3-P3 (−1419/+125), MLK3-P5 (−1960/−945), and MLK3-P6 (−296/+125) (Figure 3A). MLK3-P3 and -P6 retain proximal ERE whereas MLK3-P5 has no ERE. The 3′deletions, MLK3-P2 (−2993/−1293) and MLK3-P4 (-2993/+1937) were designed to verify the activity exclusively from distal ERE (Figure 3A). The *Renilla* expression vector, pCMV-RL, was used as an internal control for adjusting transfection efficiency. In MCF7 cells (Figure 3B), the luciferase activity of full length *MLK3* promoter (MLK3-P1) or other fragments that contain either distal ERE (MLK3-P2 and MLK3-P4) or proximal ERE (MLK3-P3 and MLK3-P6) were differentially repressed upon E2 treatment. MLK3-P5, which lacks either of EREs was unresponsive to the E2 (Figure 3B). The 3X-ERE-Luc reporter plasmid was used as a positive control for E2 response which showed induction with E2. To confirm that this repression is ER dependent, we also performed luciferase assay in ER^−^ MDA-MB-231 cells. As expected, E2 was unable to antagonize *MLK3* promoter activity with any of the promoter fragments in these cells (Figure 3C). To determine that repression of *MLK3* promoter activity was mediated via the identified ERE motifs, we mutated distal and proximal EREs separately or in combination in MLK3-P1 promoter (Figure 3D). Mutations did affect the overall activity of full length as compared to WT promoter and mutations in distal ERE significantly reduced the repressive effect of E2 on full length *MLK3* promoter, while this effect was modest when mutation was introduced in proximal ERE (Figure 3D). Furthermore, synergistic effect was observed when both EREs were mutated (Figure 3D). These data collectively suggest that the predicted EREs on the *MLK3* promoter are functional and responsive to the estrogen-mediated downregulation of *MLK3* transcripts.

### Estrogen receptor binds to the *MLK3* gene promoter

**Figure 4:**
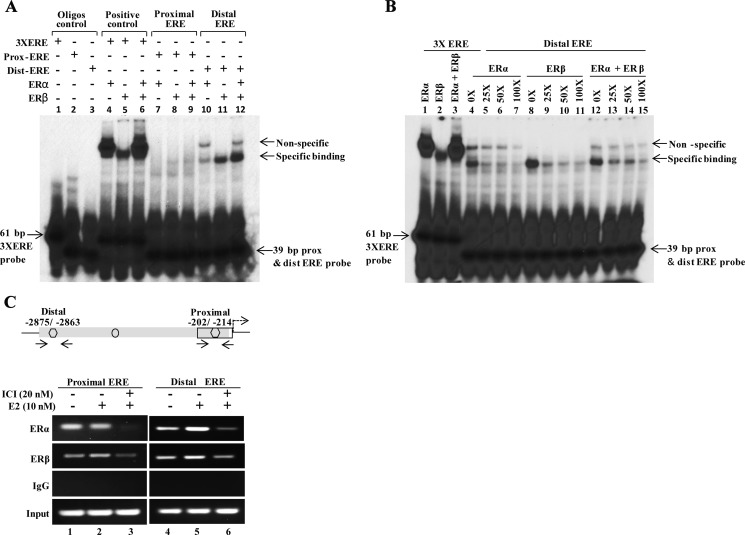
ERα and ERβ directly interact with EREs on *MLK3* promoter (**A**) In vitro translated human ERα and ERβ receptors were incubated with P^32^-labeled proximal and distal EREs oligonucleotides and complexes were separated by electrophoresis. An oligo containing 3 copies of vitellogenin gene ERE (3X ERE) in tandem was used as a positive control. Free probes are shown at the bottom of gel. (**B**) Competitive EMSA was performed to confirm specific binding of ERα (lane 4–7) and ERβ (lane 8-11) to the distal ERE. The 25-, 50- or 100-fold molar excess of unlabeled oligonucleotide were added into tubes containing labeled distal ERE. (**C**) Chromatin extracts were prepared from MCF7 cells treated with E2 (10 nM) or ICI-182, 780 (20 nM) alone or together, as indicated. Formalin fixed chromatin was immunoprecipitated with specific antibodies. Amounts of co-precipitated DNA and the corresponding amount in the input chromatin samples were measured by PCR using primers that were designed in the region flanking EREs. Co-immunoprecipitation with IgG served as a negative control.

The biological effects of estrogens are mediated by two estrogen receptors, estrogen receptor α (ERα) and estrogen receptor β (ERβ). To determine which estrogen receptor binds to the predicted EREs on *MLK3* promoter, gel mobility shift assays were performed with two EREs using *in vitro* translated ERα and ERβ proteins (Supplementary Figure 2A). The double-stranded probe, encompassing individual ERE (Supplementary Table 1), was first dephosphorylated (using calf-intestinal phosphatase) and end-labeled with T4-polynucleotide kinase using [α-^32^P]dCTP and incubated with *in vitro* translated ER proteins. The gel shift assays revealed robust ERα and ERβ binding with distal ERE but rather weaker binding with proximal ERE (Figure 4A). Furthermore, binding of ERα was weaker compared to ERβ on the distal ERE. However, when both isoforms of ER receptor were added a synergistic binding was observed (Figure 4A). The 3X ERE was also used as a positive control for the gel shift assays and as expected both ERα and ERβ proteins were bound to 3X EREs (Figure 4A). In addition, gel shift assay was also performed with labeled oligonucleotides and nuclear extracts (Supplementary Figure 2B) prepared from ER^+^ MCF7 cells. Likewise, binding was observed more prominently with distal ERE compared to proximal ERE (Supplementary Figure 2C). The interaction studies clearly suggest a functional interaction between estrogen receptors and EREs on *MLK3* promoter, whereas the distal ERE showed a higher affinity in comparison to the proximal ERE. To test the specificity of interaction between distal ERE and ER, an excess of unlabeled (cold) distal ERE oligonucleotide was added as a competing agent for radiolabeled distal ERE. The interaction between ER and distal ERE complex was partially displaced by a 50-fold molar excess of the cold probe, whereas 100-fold excess probe almost completely displaced the labeled distal ERE from ER (Figure 4B), suggesting this interaction between ER and distal ERE to be specific.

We further determined the *in vivo* recruitment of ERα and ERβ on the *MLK3* gene promoter in ER^+^ MCF7 cells by chromatin immunoprecipitation (ChIP) assays. MCF7 cells were treated either with E2 (10 nM) alone or with ER antagonist, ICI 182,780 (20 nM) compound that is reported to degrade estrogen receptor [18, 19]. The immunoprecipitated DNA by ChIP-validated anti- ERα or -ERβ antibodies were amplified using MLK3 specific ChIP primers (Supplementary Table 1). PCR amplification revealed recruitment of ERα and ERβ on both the EREs on *MLK3* promoter (Figure 4C), in agreement with our *in vitro* results. The occupancy of *MLK3* promoter by ERα and ERβ was modestly enhanced following treatment with E2, and concurrently reduced upon pretreatment with ER antagonist, ICI 182,780 compound (Figure 4C).

### E2-bound ERα recruits transcriptional corepressors on to the *MLK3* promoter

**Figure 5:**
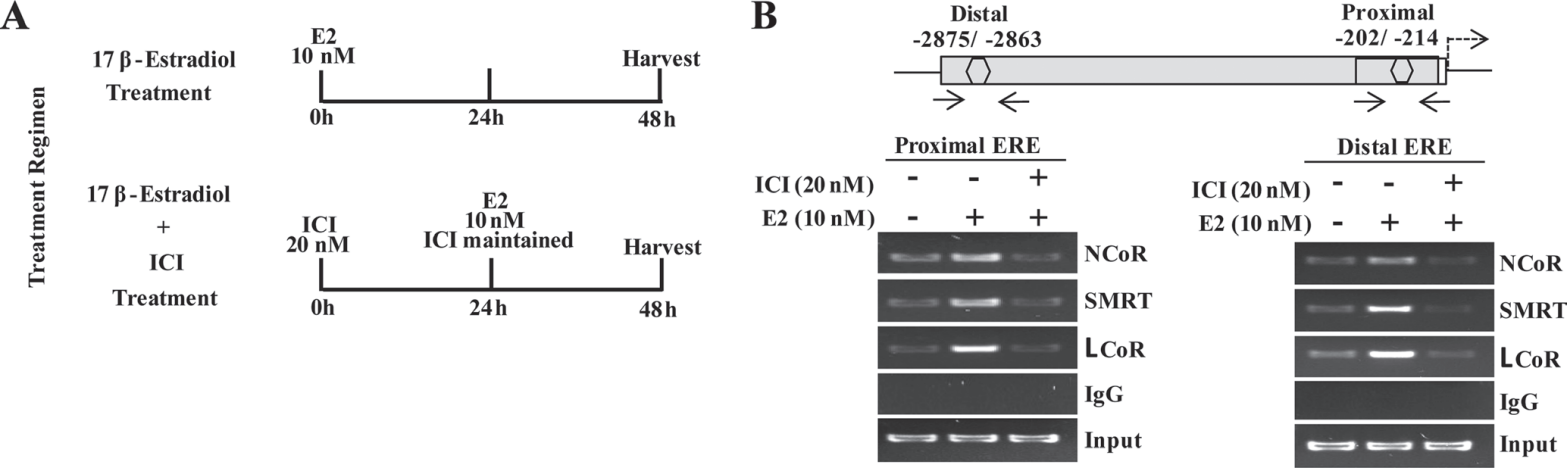
Transcriptional corepressors are recruited onto *MLK3* promoter (**A**) Treatment regimens for ChIP assays. (**B**) The primary ChIP was performed with anti-ERα antibody, and re-immunoprecipitated second time with antibodies against NcoR or SMRT or LCoR. Final immunocomplexes were eluted in elution buffer and processed for PCR.

**Figure 6:**
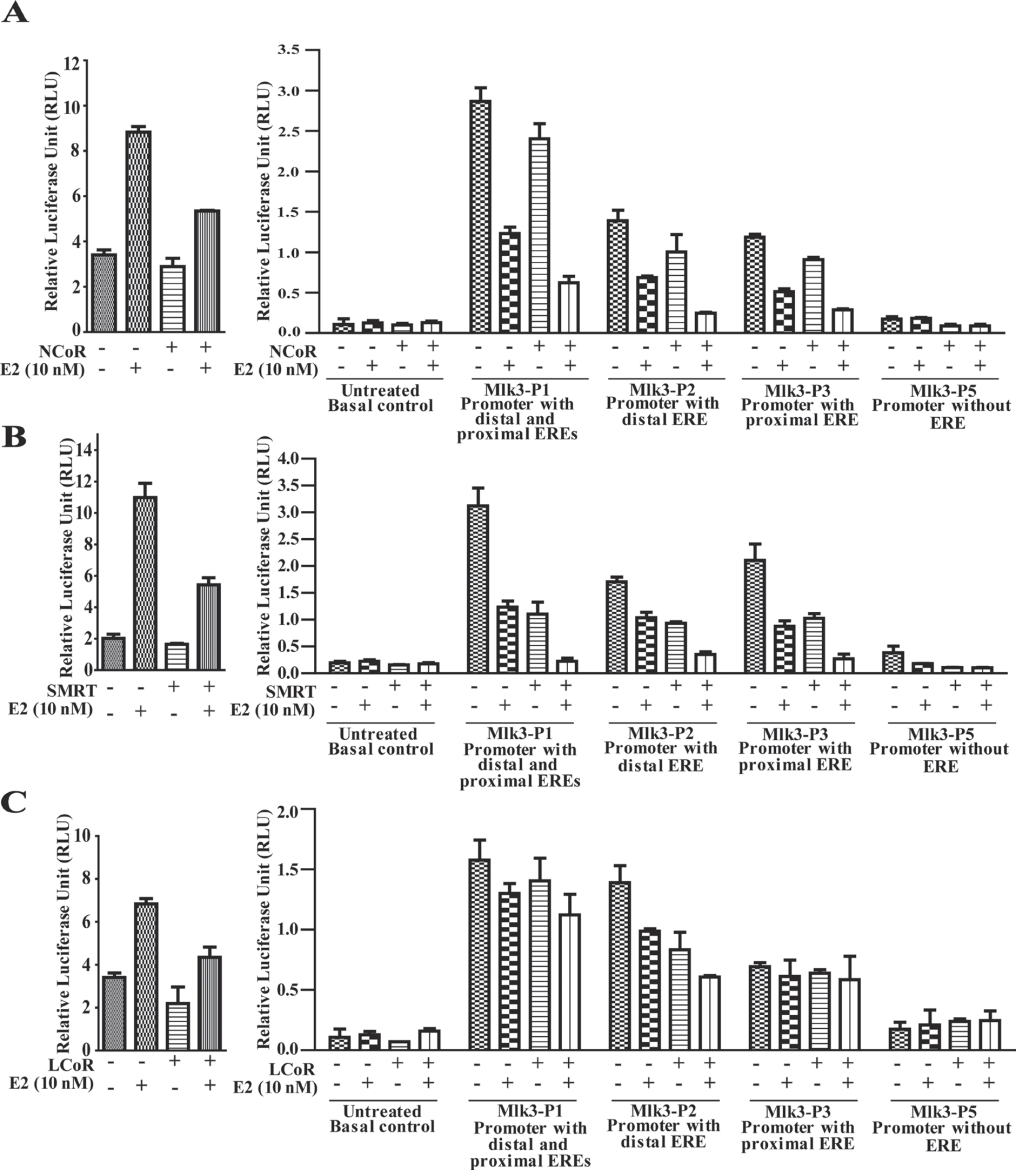
Recruitment of transcriptional corepressors onto *MLK3* promoter repress its transcriptional activity MCF7 cells were transfected individually with MLK3 promoter constructs; MLK3-P1, MLK3-P2, MLK3-P3 and MLK3-P5 in presence and absence of: (**A**) NcoR, (**B**) SMRT and (**C**) LCoR expression vectors. Wherever indicated, cells were treated with E2 (10 nM), 24 hrs. post-transfection. pGL3-basic vector containing 3XERE was used as a positive control as shown on left side of each panel. Cells were harvested 48 hrs. post-transfection and analyzed for luciferase activity and normalized with Renilla luciferase light units. The data in each set represent the mean ± SD of three independent experiments.

Collectively *in situ* hybridization, promoter assay and occupancy results indicated that perhaps the repression of *MLK3* gene might be mediated via recruitment of corepressors that are known to form complex with ligand bound estrogen receptor. It is known that ER interacts with corepressors such as Nuclear Receptor Corepressor (NcoR), Silencing Mediator of Retinoic acid and Thyroid hormone (SMRT) and Ligand dependent Corepressor (LCoR) [17, 20]. To determine whether one or more of the known corepressors facilitate the ER-mediated *MLK3* transcriptional repression, sequential ChIP and reporter assays were performed. The sequential ChIP assays were done to determine the recruitment of corepressor bound to ERα on the *MLK3* promoter. MCF7 cells were treated as per the regimen shown in Figure 5A. The chromatin from cell extracts were first immunoprecipitated using anti-ERα antibody and then re-immunoprecipitated (second time), either with antibodies against NCoR or SMRT or LCoR respectively. The results clearly indicated that at basal level, all three corepressors proteins are recruited on distal and proximal EREs of the *MLK3* promoter (Figure 5B), which was enhanced in presence of E2 and antagonized in the presence of ICI compound. The sequential ChIP assays were further validated by determining the direct effect of corepressor on *MLK3* promoter activity. The MCF7 cells were co-transfected with individual corepressor along with different *MLK3* promoter constructs, as shown in Figure 6, and treated with E2. All promoter fragments containing *MLK3* ERE were repressed in presence of overexpressed corepressors and were further inhibited upon E2 treatment (Figure 6A–6C). The repressive activities of corepressors were specific because the promoter lacking ERE (i.e. MLK3-P5) did not show any activity (Figure 6A–6C). Taken together, our results clearly suggest that E2-ER axis suppresses *MLK3* transcription via recruitment of corepressors.

## DISCUSSION

Cancer cells adapt to augment their pro-survival machinery and concomitantly suppress pro-apoptotic pathway for their survival and proliferation. Specifically in breast cancer, ER is well accepted as a master regulator of gene transcription as demonstrated by studies using cell culture and animal models [21]. The ER imparts its downstream effects by both non-genomic [22, 23] and genomic actions [24–26]. The non-genomic action is an early event where various signaling pathways are regulated for cell survival, whereas genomic action is due to the prolong actions of estrogen by which various cell survival genes are turned on or pro-survival genes are repressed.

Previously, we reported that pro-apoptotic MLK3 kinase activity was repressed via non-genomic action of estrogen in ER^+^ breast cancer tumors and cell lines [7]. We reported that estrogen-induced AKT directly phosphorylate MLK3 on Ser674 site that significantly inhibit MLK3 kinase activity, leading to ER^+^ breast cancer cell survival. During the course of these experiments, we also observed that MLK3 protein expression was noticeably lower in ER^+^ tumors and cell lines, compared to ER^−^ counterparts. These observations led us to believe that MLK3 might also be repressed at the transcriptional level to block its pro-apoptotic function in ER^+^ breast cancer cells. These results also pointed to a paradoxical function of MLK3 because despite higher expression of MLK3 in ER^−^ breast tumors and cell lines, they do not endure cell death rather their proliferation and growth rates are much higher. We are currently unsure why despite a higher expression of MLK3 in ER^−^ breast tumors it does not promote cell death. One likely possibility behind this paradox might be that the downstream targets of MLK3 in ER^−^ breast tumors are unique as compared to ER^+^ tumors (unpublished results from our ongoing studies).

In this report we show that pro-apoptotic kinase, MLK3 is a direct target of E2-ER axis and its transcription is repressed by ER's genomic action. We cloned the promoter of *MLK3* that contains two classical EREs. ChIP and luciferase reporter assays clearly showed that ERα and ERβ both bind to *MLK3* promoter and the *MLK3* is transcriptionally repressed by estrogen via ER binding to ERE on the *MLK3* promoter.

Thus far, several mechanisms have been reported for E2-mediated gene repression including physiological sequelching of co-factors, direct action of co-repressor (NCoR, LCoR and SMRT) accompanied by histone deacetylation. Most of these studies are based on over expression experiments. Our studies using endogenous co-repressor with specific *MLK3* ERE showed recruitment of NCoR, SMRT and LCoR on *MLK3* transcriptional suppression. These results were further confirmed by reporter assays, indicating that these corepressors form complex with ligand bound ER to repress *MLK3* transcription. Interestingly our results also showed that the transcriptional repression of *MLK3* is also reflected at lower MLK3 protein expression in ER^+^ breast cancer cells. Therefore, it appears that E2-ER axis not only suppresses MLK3 via post-translational modification, rather it also acts through transcriptional suppression to provide survival advantage to ER^+^ breast cancer cells.

**Figure 7:**
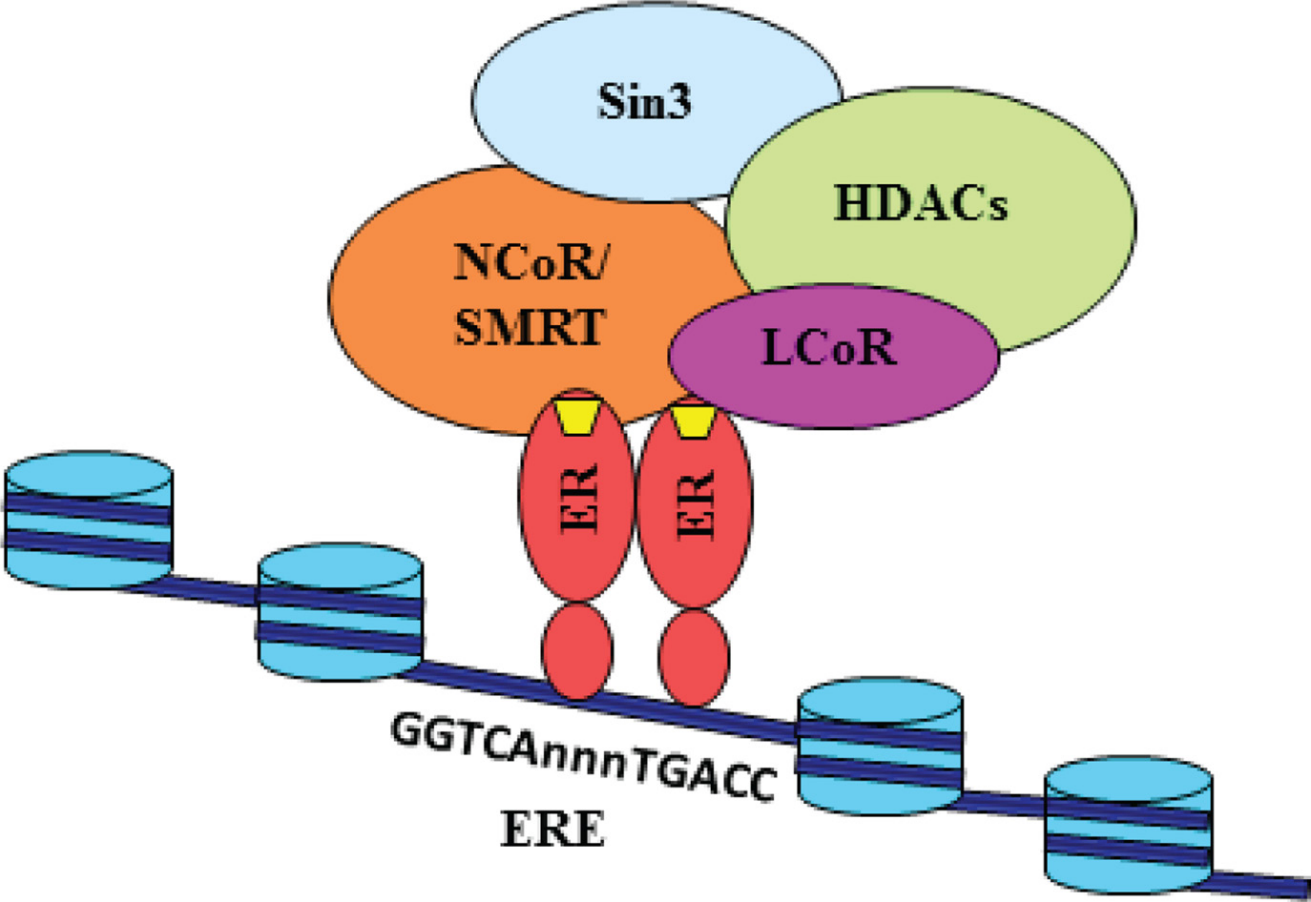
Model for the transcriptional repression of *MLK3* gene Our data suggests that E2 (yellow trapezium) binds with the estrogen receptor and instead of recruiting coactivators, engages corepressors such as NCoR, SMRT and LCoR on the promoter of Mlk3 gene. These corepressors may interact directly with Sin3, which is known to associate with HDACs. The associated corepressors/HDAC complex acts on chromatin structure by deacetylation of the histone tails, leading to its reorganization into a repressed state. The repressed state possibly blocks the recruitment of other factors, including RNA PolII.

Based on our current data and published results, we propose a model of *MLK3* gene repression via recruitment of corepressor complex containing NCoR/SMRT/LCoR on estrogen receptor, directly bound to EREs on the *MLK3* promoter (Figure 7). Our data showed recruitment of stable corepressor complex on *MLK3* ERE in the presence of E2 (Figure 5B). It is reported that corepressors (SMRT/NCoR/LCoR) recruit histone deacetylases (HDACs) either directly or through their interaction with Sin3. It is also possible that many other proteins might also be present in this complex, whose exact identity is still not known. It is also reported that deacetylation of histone tails leads to chromatin compactation and transcriptional repression. These results collectively suggest that MLK3 promoter favors recruitment of corepressors instead of coactivators to downregulate *MLK3* gene expression and finally leading to suppression of apoptotic function of *MLK3* in ER^+^ breast cancer.

In conclusion our data provide a mechanistic insight towards a novel regulation of MLK3 gene expression in ER^+^ breast cancer. Our current and published results place MLK3 as one of the key downstream molecules in E2-mediated breast cancer cell survival pathways and identification of specific mediators that antagonize its expression might serve as feasible targets to promote cell death in ER^+^ breast cancer cells.

## MATERIALS AND METHODS

### Promoter cloning

The DNA fragment (3.1 kb), containing the *MLK3* promoter was amplified from the HEK293 genomic DNA using the Expand High Fidelity PCR system (Roche Applied Science) and designated MLK3-P1. Five different deletion fragments (Figure 3A) were also generated using MLK3-P1 as a template and sub-cloned into pGL4.16 [luc2CP/Hygro] vector (Promega). The 3x-ERE-Luc reporter contains three copies of the vitellogenin ERE in the pGL2 vector was a kind gift from Dr. Janardan K. Reddy (Northwestern University, Chicago)). Promoter with mutations (single or multiple) were generated using QuickChange Multi-Site Directed Mutagenesis kit (Agilent technologies). Primer sequence used to generate mutational promoter clone are listed in the Supplementary Table 1.

### Promoter sequence retrieval and computational analysis

From the NCBI BLAST Human Genome Sequence data base, we selected 3118 bp of sequence upstream to the translation initiation codon (ATG) of the *MLK3* gene, which also includes a 125 bp coding sequence (GenBank accession number NM_002419.3). Transcription factor binding site analysis was performed using MatInspector tool from Genomatix (www.genomatix.de) and putative EREs in the *MLK3* promoter were detected.

### Nonradioactive *in situ* hybridization of paraffin sections

Tissue Micro Array (TMA) containing 16 cases of breast cancer was obtained from BioChain (Cat # Z7020007). The DNA fragment comprising partial 3′-end of *MLK3* coding sequence and partial 3′UTR was amplified from the MDA-MB-231 and MCF7 cells and cloned in to the pGEM-T vector. DIG labeled antisense and sense RNA probes were synthesized from T7 and SP6 polymerase respectively (Supplementary Figure 1B–1C). All steps prior to and during hybridization were conducted under RNase-free conditions and in-situ hybridization was done as per the manufacturer protocol (BioChain; Cat # K2191020). Briefly, Sections were deparaffinized and treated with proteinase K (2 μg/ml) pH 7.4 at room temperature for 15 min. The slides were incubated with pre-hybridization buffer for 3–4 hrs. at 50°C followed by hybridization with DIG labeled probes for 12 hrs. at 45°C. The slides were washed with SSC buffers, blocked and incubated overnight with AP-conjugated anti-digexigenin antibody. Finally, the slides were washed with PBS and incubated with NBT/BCIP solution for 4–6 hrs.

### H&E staining

Duplicate TMA slides (BioChain, Cat # Z7020007) were deparaffinized and hydrated sequentially by dipping in 100% xylene for 5 mins, 100% ethanol for 2 mins, 95% ethanol for 2 mins, 70% ethanol for 2 mins and finally into the distilled water. The slides were stained with hematoxylin stain 2 (Thermo Fisher) for 4 mins and washed under running tap water followed by one quick dipping in ammonia water and incubated in eosin y (1% alcoholic solution) for 1 minute. Finally, the slides were dehydrated by dipping in serial ethanol solutions (70–100%) followed by a dip in xylene.

### Cell treatment, transient transfection and luciferase assay

The ER^+^, MCF7, ZR75.1 and T47D, and ER^−^, MDA-MB-231 breast cancer cell lines were obtained from American Type Culture Collection (ATCC). Breast cancer isogeneic cell line pair, T47D:A18 (ERα^+^) and T47D:C42 (ERα-) were obtained from Dr. Debra A. Tonetti (University of Illinois at Chicago, Chicago, IL). These cells lines were initially created in the laboratory of Dr. V. C. Jordan [15]. Breast cancer cell lines were maintained in DMEM containing 10% fetal bovine serum (FBS), 2 mmol/L glutamine, and antibiotics (penicillin G/streptomycin). The T47D::C42 cells were grown in the medium without phenol red containing 10% CSS and 3 μg insulin.

To evaluate the estrogenic effect of exogenously added E2, the cells were cultured in DMEM without phenol red, supplemented with 5% charcoal-dextran-stripped FBS (CSS), antibiotics, and 2 mmol/L glutamine. For E2 treatment, cells were starved for 12 hrs. in DMEM without phenol red, supplemented with 0.2% CSS and treated with 10 nmol/L of E2 (Sigma) at different time intervals. For ER antagonist treatment, starved cells in phenol red free DMEM were pretreated with 20 nmol/L ICI 182,780 (Tocris Bioscience) for 24 hrs., before E2 treatment. The half-life of E2 is13-17 hrs. and therefore culture media were replenished with fresh E2 every 15 hrs. For luciferase assay, 0.4 × 10^6^ MCF7 and MDA-MB-231 cells were seeded in 24-well plate in phenol red free DMEM and 0.2% CSS. Sixteen hours later, medium was changed to the fresh starvation medium and cells were transfected using X-TremeGene HP reagent with 0.25 μg of promoter DNA constructs and 0.025 μg pCMV-RL (*Renilla* Luciferase). Luminescence of firefly and Renilla were detected on the Berthold Centro XS3 machine equipped for the Dual Luciferase Assay using Mikrowin2000 software. For control, equimolar amounts of promoter and enhancer less pGL4.16 [luc2CP/Hygro] vector was used along with pCMV-RL construct.

### RNA preparation and real time PCR

Total RNA was prepared from indicated cell lines and using TRIzol reagent (Invitrogen, Carlsbad, CA, USA). RNA quality was assessed by checking the integrity of 18S and 28S rRNA on the denaturing agarose gel after glyoxylation. The cDNA were synthesized using the Superscript III First-Strand Synthesis System kit (Invitrogen, Carlsbad, CA, USA) following manufacturer's protocols. For QPCR, the cDNA samples were diluted and amplified using the SYBR Green PCR Master Mix (Applied Biosystems). Primer sequence are listed in the Supplementary Table 1. All real-time PCR reactions were performed using the ABI StepOne Plus detection system (Applied Biosystems). The thermal cycler was programmed: 50°C for 2 min followed by an initial denaturation step at 95°C for 10 min, 40 cycles at 90°C for 10s, 60°C for 30 s finally subjecting to melting temperature to check amplification curve. The experiments were carried out in triplicate. The relative quantification in gene expression was determined using the 2^−ΔΔCt^ method using 18S rRNA as a housekeeping.

### Immunoblotting

This was performed following protocols described previously [27–29]. Briefly, cells were lysed in buffer containing, 1% NP 40, 20 mM Tris/HCl (pH 8.0), 150 mM NaCl, 2 mM EGTA (pH 8.0), 10% glycerol, 50 mM β-glycero-phosphate, 1 mM sodium orthovanadate, 1 mM DTT, 1 mM phenylmethylsulfonyl fluoride and 1X protease inhibitors cocktail (Roche). Equal amounts of protein were separated on denaturing SDS-PAGE, transferred onto polyvinylidene difluoride (PVDF) membranes and Western Blot analysis was performed by incubation with primary antibodies against: MLK3 (Epitomics; Cat # 2000-1), ER-alpha (HC-20; Santa Cruz; Cat # sc-543), ER-beta (H-150; Santa Cruz; Cat # sc-8974) and GAPDH (Santa Cruz; Cat # sc-25778). The specific signals were finally detected using HRP-conjugated secondary antibodies and developed with enhanced chemiluminescence (ECL).

### Gel shift assay with *in vitro* synthesized receptors and nuclear extract

Human ERα and ERβ were synthesized using the TNT (transcription/translation)-coupled *in vitro* system (Promega). Oligonucleotides corresponding to the distal and proximal-ERE (see Supplementary Table 1) were annealed, dephosphorylated using calf-intestinal phosphatase (CIP) and end-labeled with T4-polynucleotide kinase using [α-^32^P]dCTP. The *in vitro* synthesized ERα and ERβ were incubated individually or together at 25°C for 10 min in binding buffer containing 50 mM Tris/HCl, pH 7.5, 250 mM NaCl, 5 mM MgCl2, 5 mM EDTA, 0.5 mM dithiothreitol (DTT), 250 μg/ml poly (dI-dC), 5 μg of nonspecific salmon-sperm DNA, and 20% glycerol. Radiolabeled double stranded MLK3-EREs or positive control 3XERE probe (1 ng) were added to the reactions (total volume, 20 μl), and incubated for an additional 20 min. Reaction mixtures were analyzed by electrophoresis at 4°C on 3.5% polyacrylamide gels (30:1 acrylamide/*N*, *N*_-methylenebisacrylamide weight ratio) with 22 mM Tris base, 22 mM boric acid, 1 mM EDTA as running buffer. Nuclear extracts from E2 (10 nM) and or ICI 812, 780 (20 nM) treated (48 hrs.) MCF7 cells were prepared, using an extraction kit (Millipore, Cat # 2900) following manufacturer protocol. In brief, cells were washed with Dulbecco's modified phosphate-buffered saline without Mg^2+^ and Ca^2+^ at pH 7.4. The harvested cells were suspended in 5-pellet volumes of 0.3 M sucrose–2% Tween 40 in buffer A. After freezing, the cells were thawed and gently homogenized, and the suspension was layered onto 1.5 M sucrose in buffer A and centrifuged at 25,000 × g. Nuclei were washed with 0.3 M sucrose in buffer A, and nuclear proteins were extracted with 2.5 volumes of buffer B. Extracts were centrifuged at 100,000 × g for 1 h, and the supernatant was dialyzed for 4 h at 4°C against buffer C prior to use in electrophoretic mobility shift assays (EMSAs). Nuclear extracts were incubated with the antibody in the same 20-μl reaction volume for 30 min at 4°C before addition of the probe and processing as described above. Gels were then dried and auto radiographed.

### ChIP and sequential ChIP

Nuclei from the MCF7 cells treated with either E2 (10 nM) or ICI 182,780 (20 nM) compound were treated with 1% formaldehyde for 30 mins. to cross-link the DNA-binding proteins to cognate cis-acting elements. Nuclear homogenates were sonicated to shear the chromosomal DNA to an average length of ~1,000 bp. The chromatin was precleared by incubating with serum coupled to protein A-agarose beads, saturated with bovine serum albumin (1 mg/ml) and salmon sperm DNA (0.4 mg/ml). The ERs were immunoprecipitated overnight with ChIP validated antibodies (1–2 μg), specific to ERα (HC-20; Santa Cruz; Cat # sc-543) and ERβ (H-150; Santa Cruz; Cat # sc-8974) at 4°C. Protein-antibody complexes were pulled down with protein A beads coated with 1% bovine serum albumin and washed sequentially by low salt wash buffer I (0.1% SDS, 1% Triton X-100, 2 mM EDTA, 20 mM Tris-Cl, pH 8.1, 150 mM NaCl), buffer II (0.1% SDS, 1% Triton X-100, 2 mM EDTA, 20 mM Tris-Cl, pH 8.1, 500 mM NaCl), and buffer III (0.25 M LiCl, 1% Nonidet P-40, 1% deoxycholate, 10 mM Tris-Cl, pH 8.1), eluted and reverse cross-linked. DNA in the immune complexes was extracted and used as the template in PCR amplification, using primers listed in Supplementary Table 1. For sequential ChIP assays, cell lysates were initially incubated with anti-ERα antibody, and the immune-complexes were eluted at 37°C for 30 min in re-chip buffer (1% Triton X-100, 2 mM EDTA, 150 mM NaCl, 20 mM Tris-HCl, pH 8.1), and re-immunoprecipitated second time with antibodies against NCoR (Abcam; Cat # ab24552), SMRT (Abcam; Cat # ab24551) or LCoR (Abcam; Cat # ab48339). Final immunocomplexes were eluted in elution buffer and processed for PCR.

## SUPPLEMENTARY MATERIALS FIGURES AND TABLES


