# Supplementary material for: Transcriptional regulation of mixed lineage kinase 3 by estrogen and its implication in ER-positive breast cancer pathogenesis
Source: Oncotarget. 2017 Mar 25;8(20):33172–84. doi: 10.18632/oncotarget.16566 (PMC5464859; doi:10.18632/oncotarget.16566)
Supplement: Supplementary file 1 [file oncotarget-08-33172-s001.pdf]

Transcriptional regulation of mixed lineage kinase 3 by estrogen and its implication in ER-positive breast cancer pathogenesis

Supplementary Materials

Supplementary Table 1: List of oligos used in this study

Designation	Sequence
**Full length promoter cloning**	
hMlk3(XhoI)-F	5′-CCGCTCGAGAGCTGGGATTCACAGACTCACAAG-3′
hMlk3(HindIII)-R	5′-CCCAAGCTTGCATAACCCGCTGCCTTTGGAGAC-3′
**Deletion promoter cloning**	
hMLK3-2698(XhoI)-F	5′-CCGCTCGAGCTCCAGAACCGAGGGAAGCTC-3′
hMLK3-2101(XhoI)-F	5′-CCGCTCGAGTCGCACAGTCCCACAACTTCGCAG-3′
hMLK3-1057(HindIII)-R	5′-CCCAAGCTTCCTACCGCGCCATCACCTTTCTG-3′
hMLK3-1419(XhoI)-F	5′-CCGCTCGAGGTAGAGAACAACCTTCCGTAACAG-3′
hMLK3-1700(HindIII)-R	5′-CCCAAGCTTAGGACAGACGGAACTGGCTGCA-3′
hMLK3-1034(XhoI)-F	5′-CCGCTCGAGGCAGAAAGGTGATGGCGCGGTAG-3′
hMLK3-2049(HindIII)-R	5′-CCCAAGCTTGGAGTCTTGCTCTGTCGCCCAGG-3′
**Mutational promoter cloning**	
MLK3_D_Mut-F	5′-GTGTAGGGTTCAA***T***GTCACTC***A***AT***A***CAAGAAAGGAAGAC-3′
MLK3_D_Mut-R	5′-GTCTTCCTTTCTTG***T***AT***T***GAGTGAC***A***TTGAACCCTACAC-3′
MLK3_P_Mut-F	5′-CCCCCCGGCCT**T**A**T**CACACTGC**A**CTGGGTGCCC-3′
MLK3_P_Mut-R	5′-GGGCACCCAG**T**GCAGTGTG**A**T**A**AGGCCGGGGGG-3′
**QPCR**	
hMLK3_Q-5F	5′-GGCGAGCGTATCAGCATG-3′
hMLK3_Q-5R	5′-GGGAAAGGTGGGCGAATC-3′
hMLK3_Q-6F	5′-CCCTGTTGCTGGACCTG-3′
hMLK3_Q-6R	5′-GGAGCAGAGCGTGATGTC-3′
**EMSA**	
3X_ERE_S	5′-GTCACAGTGACCTGCGGATCTAGGTCACAGTGACCTGCGGATCCGCAG GTCACTGTGACC-3′
3X_ERE_AS	5′-GGTCACAGTGACCTGCGGATCCGCAGGTCACTGTGACCTAGATCCGCA GGTCACTGTGACC-3′
MLK3_D_ERE_S	5′-GTGTAGGGTTCAA***GGTCACTCGATCC***AAGAAAGGAAGAC-3′
MLK3_D_ERE_AS	5′-GTCTTCCTTTCTT***GGATCGAGTGACC***TTGAACCCTACAC-3′
MLK3_P_ERE_S	5′-CACCCCCCCGGCC***TGACCACACTGCC***CTGGGTGCCCTCC-3′
MLK3_P_ERE_AS	5′-GGAGGGCACCCAG***GGCAGTGTGGTCA***GGCCGGGGGGGTG-3′
**ChIP**	
MLK3_D_ChIP-F	5′-CATCACCTCTCCACACAACCAG-3′
MLK3_D_ChIP-R	5′-ACGTGGAGAGAGAGGAATCACTG-3′
MLK3_P_ ChIP-F	5′-GAAGCTCGGGTCCCTCCAAG-3′
MLK3_P_ ChIP-R	5′-CAGCCCCAGACCCACGCCTC-3′
***In-Situ* Hybridization**	
MLK3_ISH-F	5′-CATTGATCCCTGGAGCTTTGTGTC-3′
MLK3_ISH-R	5′-CAACCAGCTGGGTACAGTGTTG-3′

Designation: D: Distal, P: Proximal, F: Forward, R: Reverse, S: Sense stand, AS: Antisense strand, RE sites: Underlined, Bold italics: ERE, Bold italics and grey shading: ERE motifs with mutated nucleotides.

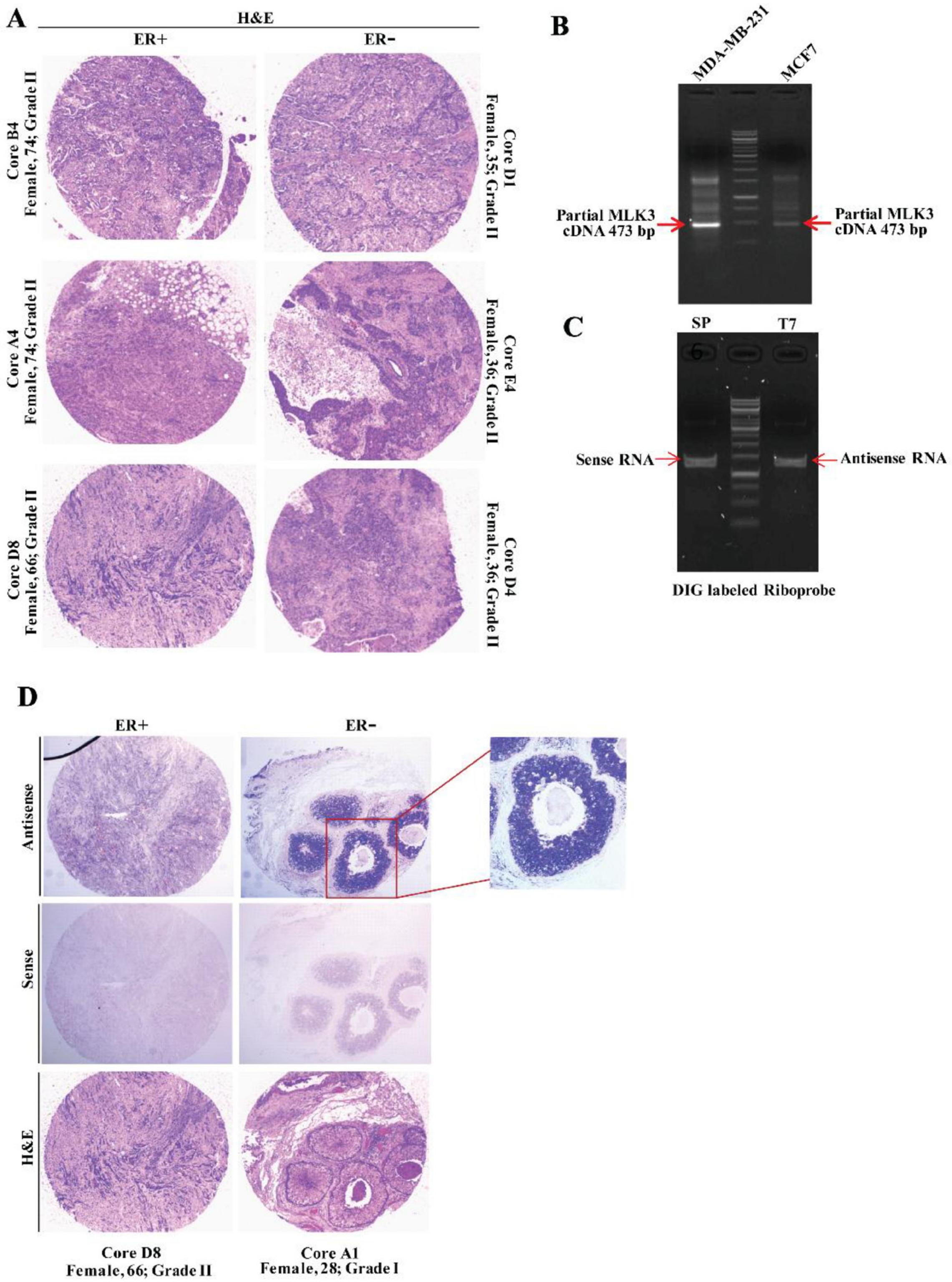

Supplementary Figure 1: (**A**) Representative photomicrographs of hematoxylin/eosin-stained breast tissue cores as shown in Figure 1B. Left panel shows cores that were obtained from ER+ breast cancer patients and panel at the right shows cores from ER^{-} patients. (**B**) PCR amplification of probes from cDNA made from MDA-MB-231 and MCF7 breast cancer cell lines used for in-situ hybridization. (**C**) Sense and anti-sense RNA probes synthesis using SP6 and T7 RNA Polymerase. (D) *In-situ* hybridization showing *MLK3* expression in ER^{-} Comedo carcinoma compared to an ER^{+} core.

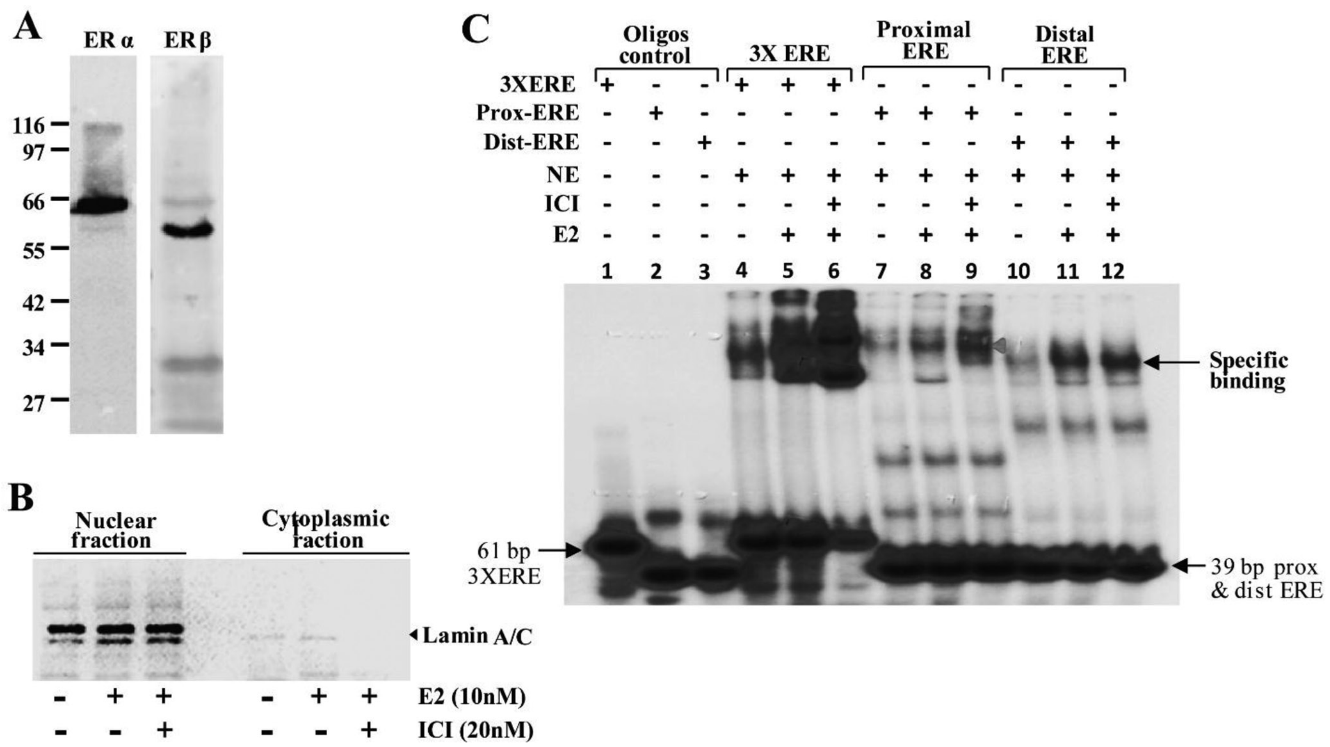

Supplementary Figure 2: (**A**) *In-vitro* translated estrogen receptors detected by western blot. (**B**) Nuclear and Cytoplasmic fractions prepared from MCF7 cells after E2 (10 nM) or ICI-182,780 (20 nM) treatments. Purity of nuclear and cytoplasmic fractions were judged by Lamin A/C. (**C**) *Mlk3* transcriptional activation by E2 is mediated via ER binding to distal and proximal-EREs. EMSA was performed with 5 µg nuclear extracts (NE) as shown in B. Endogenous ERs bind with both proximal and distal *Mlk3* EREs. Lanes 1–3 are oligos control without NE. Super shifts were observed in presence of NE and EREs: 3X consensus ERE (lanes 4–6), proximal ERE (lanes 7–9), and distal ERE (lanes 10–12). Red arrow points to the non-specific binding.
